# Inhibiting lncRNA NEAT1 Increases Glioblastoma Response to TMZ by Reducing Connexin 43 Expression

**DOI:** 10.1002/cnr2.70031

**Published:** 2024-10-25

**Authors:** Jinxing Liang, Jia‐xiu Xie, Junhui He, Yi Li, Dongmei Wei, Rongfei Zhou, Guining Wei, Xuehua Liu, Qiudan Chen, Dongmei Li

**Affiliations:** ^1^ Guangxi Key Laboratory of Traditional Chinese Medicine Quality Standards, Research Center of Traditional Chinese Medicine and Ethnic Medicine Guangxi Institute of Chinese Medicine and Pharmaceutical Science Nanning China; ^2^ Pharmaceutical College Guangxi Medical University Nanning China; ^3^ Department of Cardiology Sir Run Run Hospital of Nanjing Medical University Nanjing China; ^4^ Department of Clinical Laboratory, Central Laboratory, Jing'an District Center Hospital of Shanghai Fudan University Shanghai China; ^5^ School of Chemistry & Pharmaceutical Sciences, State Key Laboratory for the Chemistry and Molecular Engineering of Medicinal Resources Guangxi Normal University Guilin China

**Keywords:** chemotherapy sensitivity, Connexin 43, miR‐454‐3p, NEAT1, temozolomide

## Abstract

**Objectives:**

Glioblastoma multiforme (GBM) is considered the most assailant subtype of gliomas, presenting a formidable obstacle because of its inherent resistance to temozolomide (TMZ). This study aimed to characterize the function of lncRNA NEAT1 in facilitating the advancement of gliomas.

**Methods:**

The expression level of NEAT1 in glioma tissues and cells was detected by qRT‐PCR. RNA interference experiment, cell proliferation assay, FITC/PI detection assay, immunoblotting, bioinformatics prediction, a double luciferase reporter gene assay, RNA immunoprecipitation (RIP) assay, SLDT assay and correlation analysis of clinical samples were performed to explore the regulatory effects of NEAT1, miR‐454‐3p and Cx43 and their role in malignant progression of GBM. The role of NEAT1 in vivo was investigated by an intracranial tumor formation experiment in mice.

**Results:**

The results showed that recurring gliomas displayed elevated levels of NEAT1 compared to primary gliomas. The suppression of NEAT1 led to a restoration of sensitivity in GBM cells to TMZ. NEAT1 functioned as a competitive endogenous RNA against miR‐454‐3p. Connexin 43 was identified as a miR‐454‐3p target. NEAT1 was found to regulate gap junctional intercellular communication by modulating Connexin 43, thereby impacting the response of GBM cells to TMZ chemotherapy. Downregulation of NEAT1 resulted in enhanced chemosensitivity to TMZ and extended the survival of mice.

**Conclusions:**

Overall, these results indicated that the NEAT1/miR‐454‐3p/Connexin 43 pathway influences GBM cell response to TMZ and could offer a potential new strategy for treating GBM.

## 1 Introduction

Glioblastoma multiforme is the predominant malignant tumor originating in the brain and central nervous system (CNS) in adults, making up 45.2% of all malignant primary tumors. Characterized by its hostile actions, rapid cell replication, and tendency to undergo tissue decay [1, 2]. The prevailing treatment strategy for managing GBM consists of aggressive surgical resection, complemented by adjuvant chemoradiotherapy. Despite this standard protocol, the development of therapy resistance in tumor cells poses a significant obstacle to achieving successful treatment outcomes [3, 4]. Temozolomide (TMZ) chemotherapy is commonly utilized as the primary treatment option for GBM, leading to improvements in overall patient survival rates [5, 6]. Around 55% of individuals with GBM show insensitivity to TMZ because of the existence of the methylguanine methyltransferase DNA repair mechanism [7, 8]. It is crucial to fully comprehend the mechanisms that cause resistance to therapy in order to create new and effective treatment approaches.

Recent studies indicate that lncRNAs provoked in the development of tumors and show altered expression profiles in cancer according to Lieberman et al. [9] and Slack and Chinnaiyan [10]. These lncRNAs are pivotal in modulating key cellular mechanisms relevant to cancer progression, including cell cycle control, programmed cell death, and the spread of cancer cells [10, 11]. The lncRNA NEAT1 is a focus of attention due to its high expression in different types of human cancers like lung, esophageal, and gastric cancers, while being reduced in acute promyelocytic leukemias [12, 13, 14]. NEAT1 functions as a sponge for hsa‐miR‐98‐5p in the competition of endogenous lncRNAs, reducing its inhibitory impact on CTR1 and increasing the responsiveness of non‐small cell lung cancers (NSCLCs) to cisplatin [15]. Research has shown that NEAT1 is markedly upregulated in gliomas in comparison to surrounding non‐neoplastic tissues [16]. Furthermore, elevated NEAT1 levels have been correlated with decreased overall survival, increased likelihood of tumor recurrence, larger tumor size, and higher WHO grade [17]. Patients diagnosed with stage III–IV gliomas exhibiting heightened NEAT1 expression are associated with unfavorable prognostic outcomes [18]. Anyway, the precise function and mechanism of NEAT1 in the development of TMZ resistance in glioma, specifically in GBM, have not been fully elucidated despite the limited number of reports on the process.

The resistance of glioblastoma to chemotherapy is a multifaceted process that involves the participation of gap junctions mediated by connexins (Cx) [19]. Connexins exhibit both positive and negative correlations with chemosensitivity [20, 21]. Connexin 43 (Cx43), known as gap junction alpha‐1 protein (GJA1), is essential for controlling cell death, growth, and specialization [22]. New studies suggest Cx43 helps GBM cells become resistant to chemotherapy [20]. Carboxyl‐terminal Cx43 peptidomimetics demonstrated potential in addressing resistance to TMZ therapy by modulating intercellular junctional activity and downstream molecular signaling pathways between tumor cells and surrounding cells [23]. Following TMZ treatment, Cx43 was discovered to influence mitochondrial cell death pathways by controlling the levels of BAX2 and BCL2, along with the release of Cyt C from mitochondria [24]. These results suggested that targeting Cx43 may be able to increase the sensitivity of glioblastoma multiforme to TMZ. However, the specific regulatory process of Cx43 in this situation has yet to be fully understood.

This study utilized in vitro experiments to examine the influence of NEAT1 on GBM cell viability, proliferation, apoptosis, apoptosis‐related proteins, and ABCG2 expression in response to TMZ. Moreover, the study explored how NEAT1 and miR‐454‐3p interact to influence GBM response to TMZ chemotherapy by controlling Cx43 and gap junctions. Additionally, experiments were conducted in situ on brain tumors to investigate the impact of NEAT1 knockdown on tumor growth and survival. This study provides new perspectives on the role of NEAT1/miR‐454‐3p/Cx43 in the chemotherapeutic response of GBM, indicating a potential target for treating the disease.

## 2 Methods

### 2.1 Clinical Specimens

The study utilized 12 normal brain tissue samples and 67 glioblastoma tissue samples sourced from patients with traumatic brain injury and glioblastoma. Each sample underwent immediate freezing in liquid nitrogen. The diagnosis of glioma was confirmed by pathologists. These valuable samples were generously provided by the Sir Run Run Hospital of Nanjing Medical University. Moreover, this research received approval from the medical ethics committee affiliated with Sir Run Run Hospital of Nanjing Medical University.

### 2.2 Cell Culture and Treatment

Procell Life Science &Technology Co., Ltd provided U87MG and A172 human glioblastoma cell lines, human astrocytes NHA, and HEK‐293T human embryonic kidney 293T cells. Cells were verified using STR analysis and cultured in DMEM with FBS (10%), penicillin (100 units/mL), and streptomycin (100 ng/mL) from Gibco in the USA. The cells were incubated at 37°C, 5% CO_2_ environment. Cells used in the study were free from mycoplasma contamination. GenePharma in China provided lncRNA NEAT1 siRNA‐1, ‐2, ‐3, and control siRNA (si‐NC), whereas miR‐NC mimics, miR‐454‐3p mimics, miR‐454‐3p inhibitor, and miR‐NC inhibitor, pEXP‐RB‐Mam‐EGFP2‐h‐GJA1, pEXP‐RB‐Mam‐EGFP2‐h‐control, GJA1 siRNA, and NC siRNA were acquired from RIBOBIO located in China. Temozolomide (TMZ) was acquired from Sigma Aldrich (USA). Transfecting cells was performed using Lipofectamine 3000 from Thermo Fisher Scientific, USA. Subsequently, cells were exposed to varying concentrations of TMZ after 24 h of transfection.

**Table:** 

Name	Sequence (5′–3′)
NEAT1 siRNA‐1	Forward: GGGCUAAUCUUCAACUUGUTT Reverse: ACAAGUUGAAGAUUAGCCCT
NEAT1 siRNA‐2	Forward: GAUGCUGCAUCUUCUAAAUTT Reverse: AUUUAGAAGAUGCAGCAUCTT
NEAT1 siRNA‐3	Forward: GCAGGUUGAAGGGAAUUCUTT Reverse: AGAAUUCCCUUCAACCUGCTT
si‐NC	Forward: UUCUCCGAACGUGUCACGUTT Reverse: ACGUGACACGUUCGGAGAATT
miR‐454‐3p mimics	UAGUGCAAUAUUGCUUAUAGGGU
miR‐454‐3p inhibitor	UAGUGCAAUAUUGCUUAUAGGGU
shNEAT1	GATGCTGCATCTTCTAAAT
GJA1 siRNA	CCCUGGCCUUGAAUAUCAUTT
NC siRNA	AUGAUAUUCAAGGCCAGGGTT

### 2.3 Establishment of Stable Cell Lines

Lentivirus carrying NEAT1 shRNA or negative control (shNC) were conducted from OBiO Technology (Shanghai) Corp., Ltd. Stable cell lines were established in U87MG and A172 cells through lentiviral transduction and subsequent selection with puromycin.

### 2.4 Extraction of RNA and qRT‐PCR Analysis

Cells and tissues were processed to extract RNA with Trizol reagent from Thermo Fisher Scientific, USA, and cytoplasmic and nuclear RNA was isolated using the Cytoplasmic & Nuclear RNA Purification Kit from NORGEN, USA. RNA from total, cytoplasmic, or nuclear samples was reverse transcribed using the PrimeScript RT Reagent Kit (Vazyme Biotech Co., Ltd., China). Reverse transcription analysis of miR‐454‐3p, miR‐326, and miR‐520c‐3p expression levels was conducted using Mir‐X miRNA First‐Strand Synthesis Kits from TaKaRa in Japan. U6 expression acted as an endogenous control. The LightCycler480II Real‐Time PCR thermocycler (Roche, Switzerland) was used to analyze gene expression levels using ChamQ SYBR qPCR Master Mix (Vazyme Biotech Co., Ltd., China) [25]. GAPDH was the internal control.

**Table:** 

Name	Primer of genes (5′–3′)
NEAT1‐Forward	TGGCTAGCTCAGGGCTTCA
NEAT1‐Reverse	CCTAGTCTCCTTGCCAAGCTTC
GAPDH‐Forward	CCACCCATGGCAAATTCCATGGCA
GJA1‐Forward	TGGTAAGGTGAAAATGCGAGG
GJA1‐Reverse	GCACTCAAGCTGAATCCATAGAT
GAPDH‐Reverse	TCTAGACGGCAGGTCAGGTCCACC
miR‐454‐3p	GGGTGCAATATTGCTTATAGGGTA
miR‐326	TGGGCCCTTCCTCCAGAA
miR‐520c‐3p	GCAGTGCTTCCTTTTAGAGGGTA
U6‐Forward	GGAACGATACAGAGAAGATTAGC
U6‐Reverse	TGGAACGCTTCACGAATTTGCG

### 2.5 Cell Proliferation Assay

In 96‐well plates, 3000 cells were grown in separate wells. Cell proliferation was measured using a CCK8 kit from Dojindo Laboratories, Japan at specific time intervals according to the provided guidelines.

### 2.6 Apoptosis Detection Assay

Using the Annexin V‐FITC/PI Apoptosis Detection Kit (Vazyme, China) as directed, Annexin V and propidium iodide (PI) were labeled. Cell analysis was conducted using the Microcapillary cell analysis platform Guava easyCyte HT (Merck Millipore, USA), and data analysis was performed using GuavaSoft 3.1.1. The experiments were conducted in triplicate.

### 2.7 Protein Extraction and Immunoblotting

Cells or tissues were harvested and homogenized in RIPA buffer (Beyotime, China) on ice, with the addition of a mixture of protease and phosphatase inhibitors (Beyotime, China). After centrifuging the lysates at 12 000 rpm for 15 min at 4°C, the supernatants were isolated. Protein concentrations were quantified using the BCA assay (Beyotime, China). Subsequently, SDS‐PAGE was utilized to separate the protein samples, which were then transferred onto PVDF membranes (Roche, Switzerland) using transfer buffer (Beyotime, China). Membranes were obstructed with non‐fat milk (5%) for a period of 2 h, then incubated with primary antibodies. Signal detection was carried out using FluorChem R equipment from ProteinSimple, USA. ImageJ software was employed for the calculation of relative density.

Antibodies against Cx43 (#26980‐1‐AP), BAX (#50599‐2‐Ig), and ABCG2 (#27286‐1‐AP) were purchased from Proteintech (China). BCL2 (#CAS7511) and GAPDH (#AP0066) antibodies were purchased from Bioworld Technology, Inc. (USA).

### 2.8 Computational Analyses and Bioinformatics

The NEAT1 expression data of glioma samples from CGGA were downloaded from the Chinese Glioma Genome Atlas database (http://www.cgga.org.cn) [26]. NEAT1 expression data in TMZ‐sensitive and TMZ‐resistant cells were obtained from GSE100736 dataset in GEO database (https://www.ncbi.nlm.nih.gov/geo/query/acc.cgi?acc=GSE100736). Potential target miRNAs and genes were predicted by Starbase database (http://www.cuilab.cn/hmdd) [27], LncBase Predicted v.2 of DIANA tools (http://diana.imis.athena‐innovation.gr/DianaTools/index.php?r=tarbase/index) [28], LNCediting database (http://bioinfo.life.hust.edu.cn/LNCediting) [29], NPInter v4.0 (http://bigdata.ibp.ac.cn/npinter4), and miRDB (https://mirdb.org) [30]. miRNA and disease associations were extracted from Human microRNA Disease Database version 3.2 (HMDD v3.2) (http://www.cuilab.cn/hmdd) [31]. Venn diagrams was got from https://bioinformatics.psb.ugent.be/webtools/Venn.

### 2.9 Dual‐Luciferase Reporter Assay

The NEAT1 sequence and the 3′‐UTR of GJA1, both with anticipated miR‐454‐3p seed‐matching regions and their mutant counterparts, were created, combined, and integrated into the pMIR‐REPORTER vector from Ambion in the United States. Sequencing was used to validate the constructs. In a 24‐well plate, HEK‐293T cells were seeded and co‐transfected with the original plasmid or the altered reporter plasmid, in addition to pRL‐CMV (Promega, USA) and either miR‐454‐3p or miR‐NC for the Dual‐luciferase reporter test. Luciferase levels were assessed 24 h after transfection with the Dual Luciferase Reporter Assay System from Promega in the United States.

### 2.10 RNA Immunoprecipitation (RIP) Assay

Cells that had been transfected with miR‐454‐3p mimics or miR‐NC mimics were harvested by scraping and subsequently resuspended in ice‐cold PBS before being lysed in RIP lysis buffer obtained from Abcam, USA. The lysates were treated with a human anti‐Ago2 antibody overnight (with normal rabbit IgG as a control), followed by incubation with protein A/G beads for 1 h. After washing the beads twice, RNA was extracted and analyzed using qRT‐PCR.

### 2.11 Scrape‐Loading and Dye Transfer (SLDT)

The SLDT assay was conducted following established procedures, with a few adjustments as outlined by Xu et al. [32]. After TMZ treatment, cells transfected with si‐NEAT1 or si‐NC and pEXP‐RB‐Mam‐EGFP2‐h‐GJA1 or pEXP‐RB‐Mam‐EGFP2‐h‐control were mechanically disrupted and exposed to Lucifer Yellow CH (0.1% (w/v)) (GLPBIO, USA) for 5 min without light. Subsequent imaging was conducted using fluorescence microscopy (Leica, Germany).

### 2.12 In Situ Tumor Formation Experiment

Animal experiments approved by Ethics Committee on Laboratory Animal Management of Guangxi Institute of Chinese Medicine and Pharmaceutical Science (No. 20211104).

U87MG/shNEAT1‐Luc and U87MG/shNC cells were generated through transfection of U87MG/shNEAT1 and U87MG/shNC with luciferase. Subsequently, 1 × 10^5^ cells were suspended in 10 μL serum‐free DMEM and intracranially injected into the striatum of 6‐week‐old BALB/c nude mice using a stereotactic device with coordinates of 2 mm anterior, 2 mm lateral, and 3 mm depth from the dura. One‐week post‐implantation of tumors, mice were administered intraperitoneal injections of 20 mg/kg of TMZ in saline or an equivalent volume of physiological saline. The injections were administered daily for five consecutive days, followed by a 2‐day cessation period, constituting one cycle of TMZ treatment. This treatment regimen was repeated for a total duration of 3 weeks. In order to visualize cell proliferation, mice were administered an intraperitoneal injection of d‐luciferin (150 mg/kg) and images were captured under anesthesia using the IVIS Lumina III System (PerkinElmer, USA). Weekly image acquisition was conducted to monitor tumor growth progression. Following 3–4 weeks, the brains were perfused with 4% paraformaldehyde via cardiac perfusion and subsequently fixed with Bouin solution at 4°C overnight. The brains were then resected and subjected to H&E staining. Subsequently, the mice were euthanized to extract the tumor tissues for further analysis.

### 2.13 Statistics Analysis

Mean values with standard deviations were reported for all measurements, based on a minimum of three independent experiments, unless stated otherwise. In order to compare two groups, a Student's *t*‐test was used, while in order to compare multiple groups, a one‐way ANOVA was used. Two‐way ANOVA was utilized for comprehensive comparisons across multiple factors. The Pearson correlation coefficient was utilized for evaluating the connections among variables. Kaplan–Meier analysis with the logrank test was used to calculate the overall survival rate (OS) for comparison between groups. Data analysis was performed using either GraphPad Prism 10.1.2 software (La Jolla, USA) or IBM SPSS Statistics 25.0 software (Chicago, USA), with significance determined at a *p*‐value below 0.05.

## 3 Results

### 3.1 NEAT1 Knockdown Enhances the Sensitivity of GBM to TMZ

**FIGURE 1:**
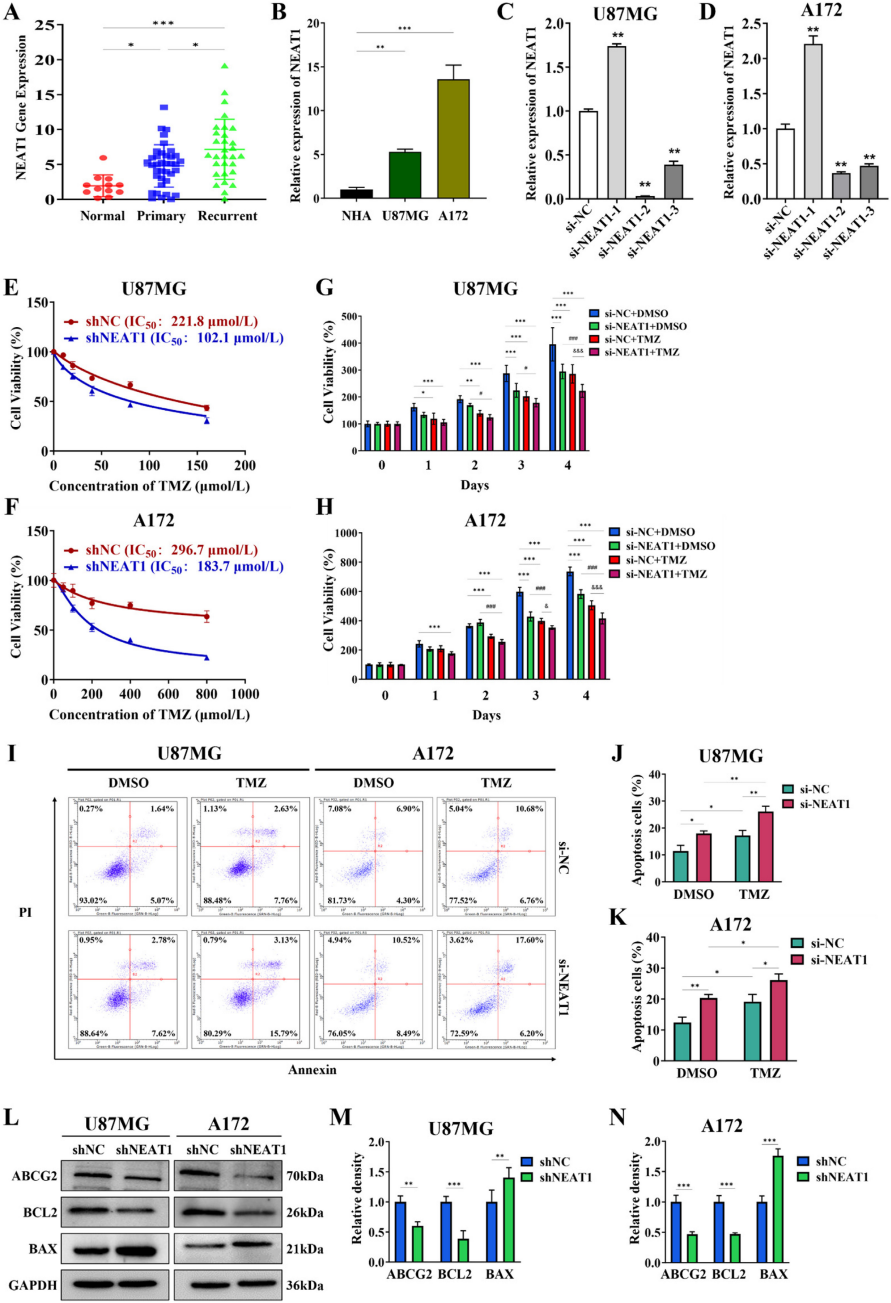
NEAT1 knockdown enhances the sensitivity of GBM to TMZ. (A) Relative expression of NEAT1 in normal brain tissues, primary and recurrent GBM tissues, normalized to GAPDH. *p < 0.05, ***p < 0.001. (B) Relative expression of NEAT1 in NHA, U87MG, and A172 cells. **p < 0.01, ***p < 0.001. (C, D) The expression of NEAT1 in U87MG and A172 cells, NEAT1 was knockdown by transfecting with si‐NEAT1‐1, si‐NEAT1‐2, or NEAT1‐3. **p < 0.01. (E, F) CCK‐8 assay analysis revealed the effect of shNC and shNEAT1 on the U87MG and A172 cells after TMZ treatment at the indicated concentrations for 48 h. (G, H) Cell proliferation assays of shNC and shNEAT1 in U87MG and A172 cells with TMZ (100 μM for U87MG cells, 180 μM for A172 cells, the same below) or equal volume DMSO treatment. *p < 0.05, **p < 0.01, ***p < 0.001, compared to si‐NC + DMSO group; ^#^ *p* < 0.05; ^###^ *p* < 0.001, compared to si‐NEAT1 + DMSO group; ^&^ *p* < 0.05; ^&&&^ *p* < 0.001, compared to si‐NC + TMZ group. (I–K) Flow cytometric analysis of si‐NC and si‐NEAT1 in U87MG and A172 cells with TMZ or equal volume DMSO treatment (48 h), *p < 0.05, **p < 0.01. (L–N) The protein levels of ABCG2, BCL2, and BAX in U87MG and A172 cells with shNEAT1 or shNC. GAPDH was used as the control. Relative density values were counted using image J software. *p < 0.05; **p < 0.01; ^#^ *p* < 0.05; ^##^ *p* < 0.01; ^&^ *p* < 0.05; ^&&^ *p* < 0.01.

We utilized qRT‐PCR to assess NEAT1 expression in clinical samples, comprising of 12 normal brain tissues, 37 primary GBM tissues, and 30 recurrent GBM tissues (insensitive to TMZ treatment). The findings revealed that recurrent glioma exhibited the highest levels of NEAT1 expression (Figure 1A), suggesting a potential association between NEAT1 and glioma prognosis. Afterward, we searched the CGGA (Chinese Glioma Genome Atlas) database to analyze the levels of NEAT1 in both initial and recurring gliomas. Consistently with our previous study, recurring gliomas displayed elevated levels of NEAT1 compared to primary gliomas (Figure S1A).

Further, we examined NEAT1 expression in human glioblastoma cells (U87MG and A172) and human astrocytes (NHA). NEAT1 expression was markedly increased in glioblastoma cells compared to NHA (Figure 1B). Furthermore, NEAT1 exhibited higher levels in the TMZ‐resistant cells when compared to the TMZ‐sensitive cells in the GSE100736 dataset (Figure S1B), which dataset illustrates the expression patterns of lncRNA and mRNA in TMZ‐resistant and TMZ‐sensitive cell lines.

To determine the possible importance of NEAT1 in influencing resistance to TMZ, NEAT1 levels were reduced by introducing si‐NEAT1‐1, si‐NEAT1‐2, and si‐NEAT1‐3 into U87MG and A172 cells (Figure 1C,D). Since the si‐NEAT1‐2 reduced NEAT1 expression most significantly in both cell lines, si‐NEAT1‐2 was used to silence NEAT1 expression in subsequent experiments. Knocking down NEAT1 (shNEAT1) in U87MG and A172 cells resulted in a heightened reaction to TMZ, demonstrated by a lower IC_50_, in comparison to the control group (shNC) (Figure 1E,F). TMZ at 100 and 180 μM were used as subsequent administration concentration in U87MG and A172 cells.

Subsequently, CCK8 assays were performed to assess the influence of NEAT1 expression on cellular proliferation in two distinct cell lines exposed to either TMZ or DMSO. The findings showed that TMZ effectively suppressed cell growth, particularly in the shNEAT1 group, when compared to the shNC group after receiving TMZ treatment (Figure 1G,H). These findings suggest that downregulation of NEAT1 may augment the susceptibility of GBM cells to TMZ. Additionally, cell apoptosis analysis was implemented in GBM cells treated with TMZ or DMSO. The results showed that rates of apoptosis were the most significantly increased in the si‐NEAT1 groups with TMZ treatment (Figure 1I–K), revealing knockdown of NEAT1 could promote the sensitivity of GBM cells to TMZ. To further investigate whether NEAT1‐mediated resistance to TMZ involves drug‐resistant protein ABCG2, apoptosis‐related protein BCL2, and BAX, Western blot analysis was conducted. The results exhibited a significant downregulation of ABCG2 and BCL2 expression upon silencing NEAT1, while an upregulation in BAX expression was observed at the protein level in shNEAT1 group when compared to the shNC group, in U87MG and A172 cells (Figure 1L–N). In summary, the results indicated that lncRNA NEAT1 exhibited high levels of expression in recurrent GBM tissues and cell lines, leading to decreased sensitivity of GBM cells to TMZ.

### 3.2 NEAT1 Acts as a Molecular Sponger for miR‐454‐3p

**FIGURE 2:**
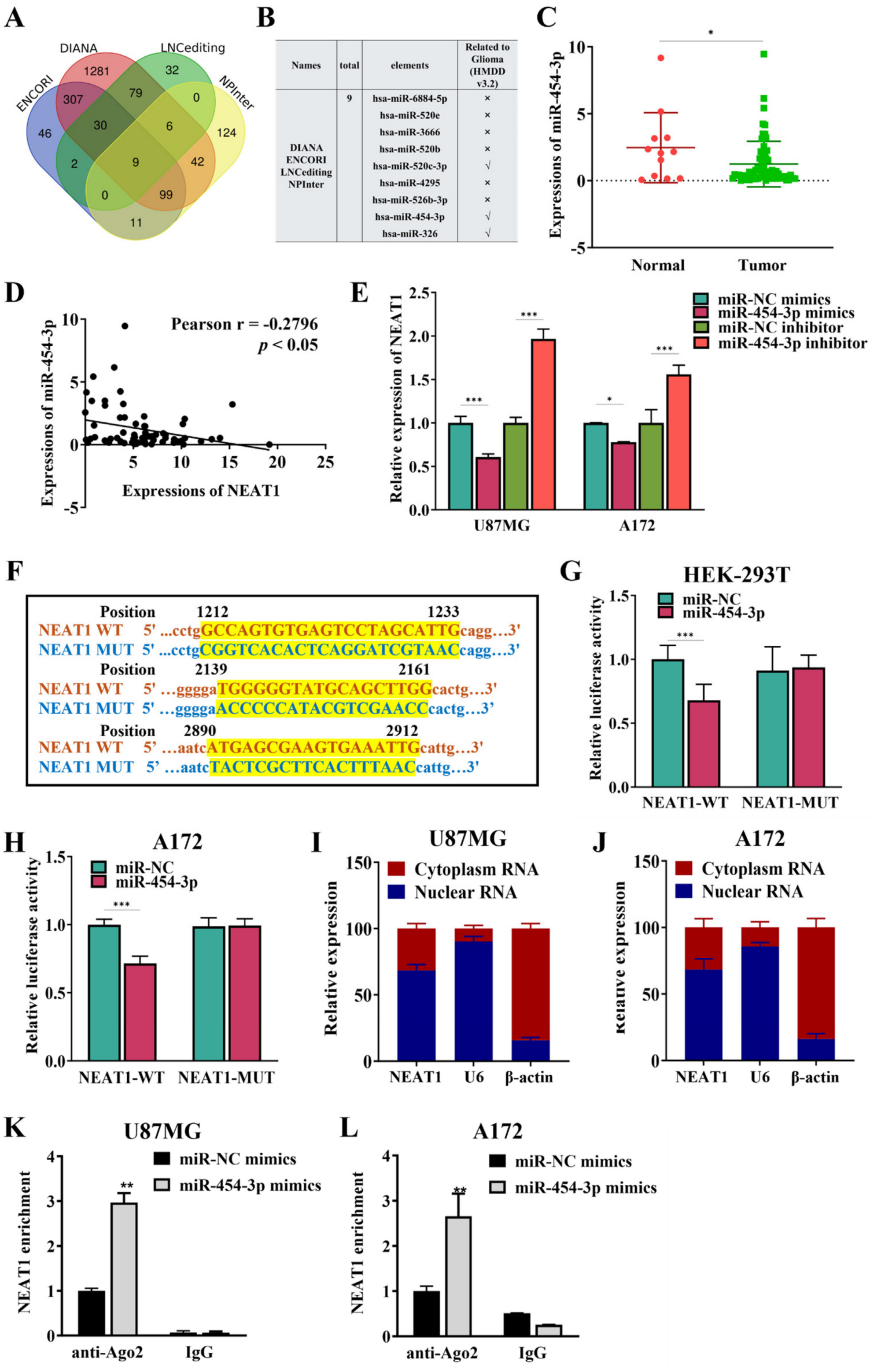
NEAT1 acts as a molecular sponger for miR‐454‐3p. (A) Venn diagram was used to achieve the potential miRNAs that may target the binding region of NEAT1. (B) Relative expression of miR‐454‐3p in normal brain and GBM tissues, normalized to U6. (C) The Pearson's correlation analysis was performed to analyze the correlation between NEAT1 and miR‐454‐3p. (D) The mRNA expression level of NEAT1. (E) Schematic illustration of the predicted binding sites between NEAT1 and miR‐454‐3p, and mutation of potential miR‐454‐3p binding sequence in NEAT1. (F, G) Dual luciferase assay was applied to assess the seed‐matching sites or mutant sites between NEAT1 and miR‐454‐3p in HEK‐293T cells and A172 cells. (H–J) QRT‐PCR was employed to analyze the expression of NEAT1 in nuclear and cytoplasmic in U87MG and A172 cells. (K, L) Anti‐Ago2 RIP analyses were applied in U87MG and A172 cells transfected with miR‐NC mimics or miR‐454‐3p mimics, the enrichment of NEAT1 was detected by qRT‐PCR. (K, L) *p < 0.05; **p < 0.01; ***p < 0.01.

Increasing proof suggests that lncRNAs can regulate target gene expression by acting as ceRNAs that trap miRNAs. Using the Starbase database, LncBase Predicted v.2 of DIANA tools, LNCediting database, and NPInter v4.0, potential miRNAs with the binding site of NEAT1 were predicted to understand the molecular mechanisms behind NEAT1's role in conferring TMZ resistance. There were nine miRNAs were selected by Venn diagrams. From the HMDD v3.2 database, we identified miR‐520c‐3p, miR‐454‐3p, and miR‐326 as being associated with glioma (Figure 2A). In GBM tissues, miR‐454‐3p exhibited down‐regulation compared to normal brain tissues (Figure 2B). Moreover, a negative correlation was found between the levels of NEAT1 and miR‐454‐3p expression in GBM tissue samples, with a Pearson correlation coefficient of −0.2796 (Figure 2C), while miR‐520c‐3p and miR‐326 were not significantly correlated with NEAT1 (Data not showed). The expression of NEAT1 was found to be significantly decreased following transfection with miR‐454‐3p mimics, and conversely increased following transfection with miR‐454‐3p inhibitors, in U87MG and A172 cells (Figure 2D), indicating that miR‐454‐3p negatively regulates lncRNA NEAT1 either directly or indirectly. There are three potential binding sites of miR‐454‐3p on NEAT1, located at regions 1212–1233, 2139–2161, and 2890–2912. Luciferase plasmids were created that included wild type NEAT1 sequences along with potential binding sites (NEAT1‐WT) and mutational versions (NEAT1‐MUT) (Figure 2E). To determine the interaction between NEAT1 and miR‐454‐3p, we performed dual‐luciferase gene reporter assays. In HEK‐293T and A172 cells, the luciferase activity of NEAT1‐WT was reduced by miR‐454‐3p, while the activity of NEAT1‐MUT remained unaffected (Figure 2F,G). Additionally, qRT‐PCR analyses revealed that NEAT1 is present in both nuclear and cytoplasmic fractions in U87MG and A172 cells (Figure 2H,I). We performed RIP assays to verify their presence in the anticipated RISC complex. Ago2 was bound to NEAT1 and miR‐454‐3p (Figure 2J,K). Our research as a whole indicates a suppressive connection between NEAT1 and miR‐454‐3p in GBM cells and tissue specimens, proposing a mutual inhibition feedback mechanism between NEAT1 and miR‐454‐3p.

### 3.3 miR‐454‐3p Targets GJA1 (Cx43)

**FIGURE 3:**
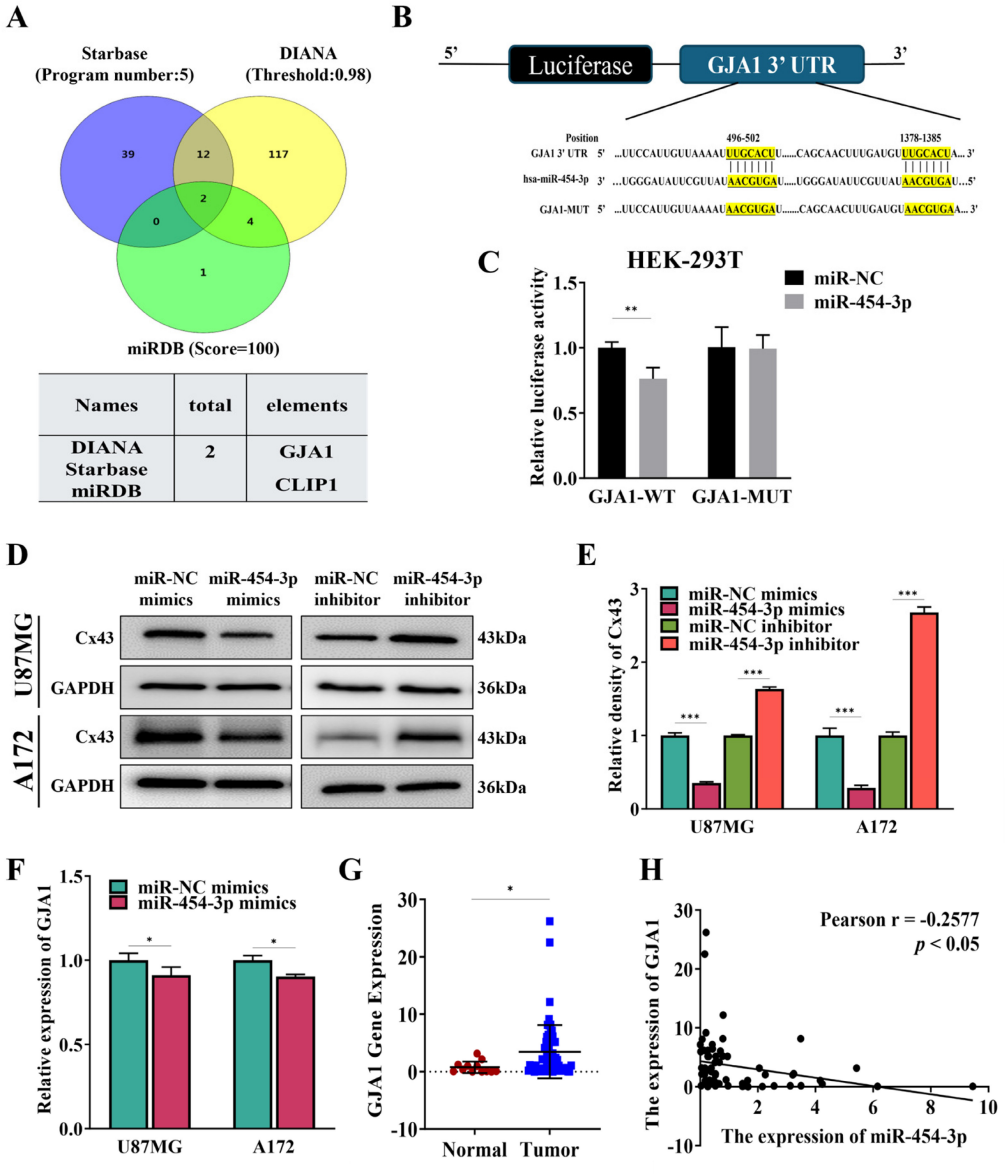
miR‐454‐3p targeted GJA1 (Cx43). (A) Venn diagram was used to achieve the potential genes that miR‐454‐3p may target the binding region. (B) Schematic illustration of the predicted binding sites between GJA1 and miR‐454‐3p, and mutation of potential miR‐454‐3p binding sequence in GJA1. (C) Dual luciferase assay was applied to assess the seed‐matching sites or mutant sites between GJA1 and miR‐454‐3p in HEK‐293T. (D, E) The protein levels of Cx43 in U87MG and A172 cells transfected with miR‐454‐3p mimics, miR‐NC mimics or miR‐454‐3p inhibitor, miR‐NC inhibitor. GAPDH was the control. Relative density values were counted using image J software. (F) The mRNA expression level of GJA1 transfected with miR‐NC mimics or miR‐454‐3p mimics. (G) Relative expression of GJA1 in normal brain and GBM tissues, normalized to GAPDH. (H) The Pearson's correlation analysis was performed to analyze the correlation between GJA1 and miR‐454‐3p. *p < 0.05; **p < 0.01; ***p < 0.01.

Starbase, DIANA, and miRDB software identified a match between the seed sequence of miR‐454‐3p and the 3′‐UTR regions of GJA1 and CLIP1 (Figure 3A). Previous studies have shown the important role of GJA1 in the resistance mechanisms of GBM [33]. As a result, we developed luciferase reporter plasmids that included both the wild type (WT) and mutant (MUT) forms of the GJA1 3′‐UTR for further investigation (Figure 3B). Subsequently, co‐transfection experiments were conducted by introducing these luciferase reporter plasmids along with miR‐454‐3p. The findings showed that increasing the expression of miR‐454‐3p resulted in a notable decrease in luciferase activity regulated by the GJA1 3′‐UTR, without affecting the transcriptional activation of the mutant GJA1 3′‐UTR reporter activity (Figure 3C). The findings strongly suggest that miR‐454‐3p interacts with GJA1 by attaching to the seed sequence located in the 3′‐UTR region. Moreover, overexpression of miR‐454‐5p resulted in decreased protein levels of Cx43 (encoded by GJA1 gene) in both U87MG and A172 cells; conversely, inhibition of miR‐454‐5p increased Cx43 protein expression levels in these cell lines as well (Figure 3D,E). Similarly, miR‐454‐3p mimics upregulated the mRNA level of GJA1 (Figure 3F).

To establish clinical relevance between miR‐454‐5p expression levels and GJA1 abundance, we examined their correlation using samples from GBM patients alongside normal brain tissues. Figure 3G shows an increase in GJA1 expression in GBM tissues compared to normal brain tissues. Furthermore, the correlation analysis conducted on samples of glioblastoma revealed a negative correlation between GJA1 levels and miR‐454‐5p expression levels, with a Pearson r value of −0.2577 (Figure 3H). Collectively, our findings provide compelling evidence supporting direct targeting of Cx43 by miR‐454‐5p in GBM.

### 3.4 NEAT1‐Mediated TMZ Resistance Mediated by Cx43

**FIGURE 4:**
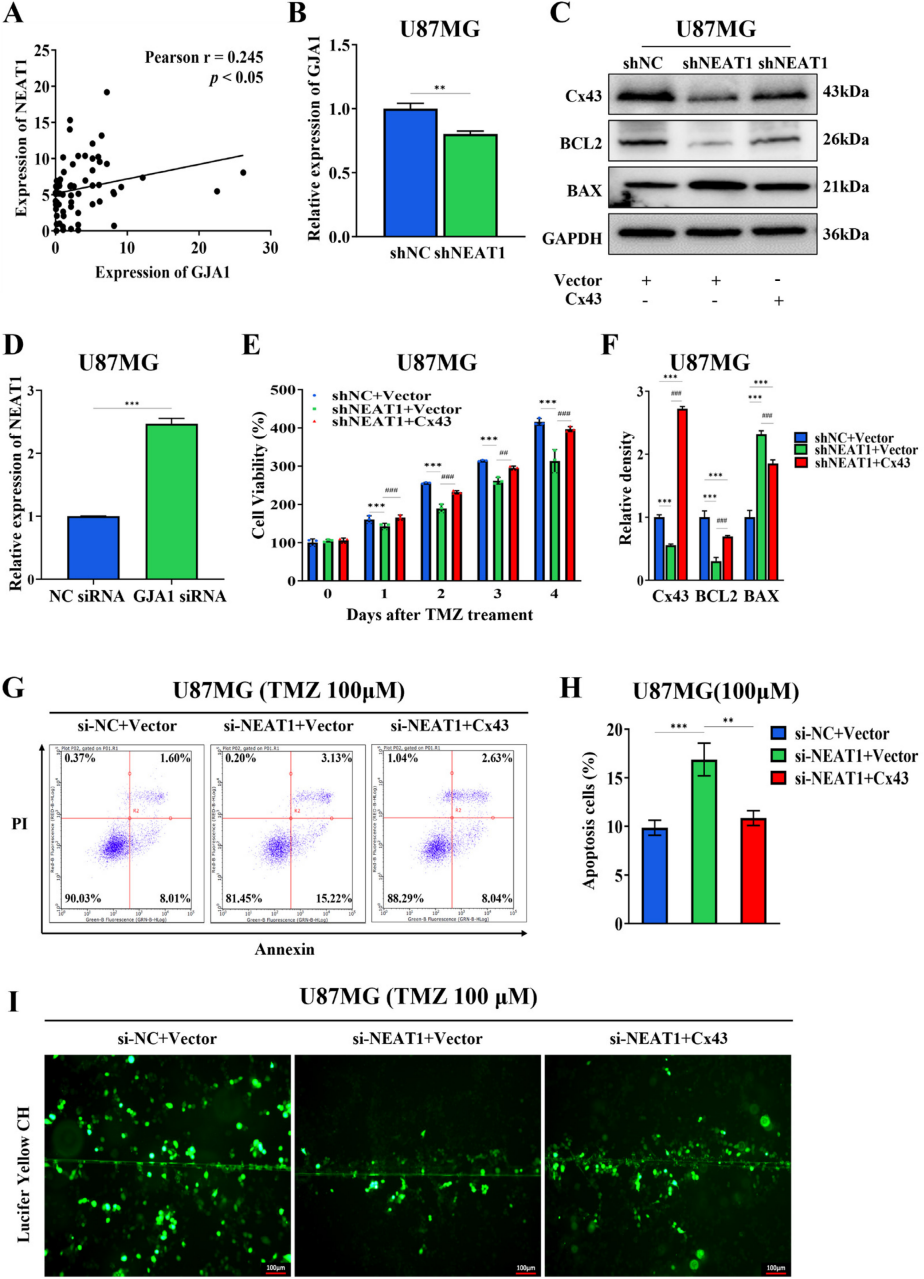
Cx43 is responsible for NEAT1‐mediated TMZ resistance. (A) The Pearson's correlation analysis was performed to analyze the correlation between NEAT1 and GJA1. (B) The mRNA expression level of GJA1 in U87MG cells with shNC and shNEAT1 group, **p < 0.01. (C, F) The protein levels of Cx43, BCL2, and BAX in U87MG cells with shNC + Vector, shNEAT1 + Vector or shNEAT1 + Cx43. ***p < 0.001, compared to shNC + Vector group; ^###^ *p* < 0.001, compared to shNEAT1 + Vector group. (D) The mRNA expression level of NEAT1 transfected with GJA1 siRNA or NC siRNA. ***p < 0.01. (E) Cell proliferation assays of shNC and shNEAT1 in U87MG and A172 cells transfected with Cx43 plasmid or Vector, with TMZ treatment. ***p < 0.001, compared to shNC + Vector group; ^##^ *p* < 0.01, ^###^ *p* < 0.001, compared to shNEAT1 + Vector group. (G, H) Flow cytometric analysis of si‐NC and si‐NEAT1 in U87MG and A172 cells transfected with Cx43 plasmid or vector, with TMZ treatment (100 μM, 48 h). **p < 0.01, ***p < 0.001. (I) The SLDT assay was conducted to detect the influence of shNC + Vector, shNEAT1 + Vector or shNEAT1 + Cx43 on gap junction intercellular communication, which was indicated by the dye spreading area. Scale bar = 100 μm.

An association between GJA1 and NEAT1 expression in GBM samples was observed in the analysis of glioblastoma samples, showing a Pearson correlation coefficient of 0.245 (Figure 4A). ShNEAT1 decreased the mRNA level of GJA1 (Figure 4B). The mRNA expression level of NEAT1 transfected with GJA1 siRNA was upregulated compared to transfected with NC siRNA (Figure 4D). In order to examine the involvement of Cx43 in NEAT1‐related biological functions that leads to resistance to TMZ in GBM, we performed experiments where Cx43 was overexpressed in NEAT1‐silenced cells (shNEAT1) and negative control cells (shNC). ShNEAT1 decreased the protein level of Cx43, and overexpression of the GJA1 plasmid resulted in the restoration of the expression level of cx43 in the shNEAT1 group (Figure 4C,F). CCK8 assays indicated that the increased expression of Cx43 effectively counteracted the inhibition of tumor cell growth and sensitivity to chemotherapy caused by NEAT1 knockdown (Figure 4E). Similarly, Cx43 overexpression reversed NEAT1 knockdown‐mediated apoptosis in si‐NEAT1 cells treated with TMZ (Figure 4G,H). Overexpression of Cx43 restored NEAT1 silencing attenuated BCL2, and decreased BAX expression aroused by shNEAT1 in U87MG cells with TMZ treatment (Figure 4C,F). Further, SLDT assay showed that NEAT1 knockdown impaired the gap junction intercellular communication, which was ameliorated by Cx43 overexpression (Figure 4I). Collectively, the results demonstrated that Cx43 was participated in the process of regulating NEAT1‐mediated TMZ resistance in GBM cells.

### 3.5 NEAT1 Depletion Enhances TMZ Sensitivity In Vivo

**FIGURE 5:**
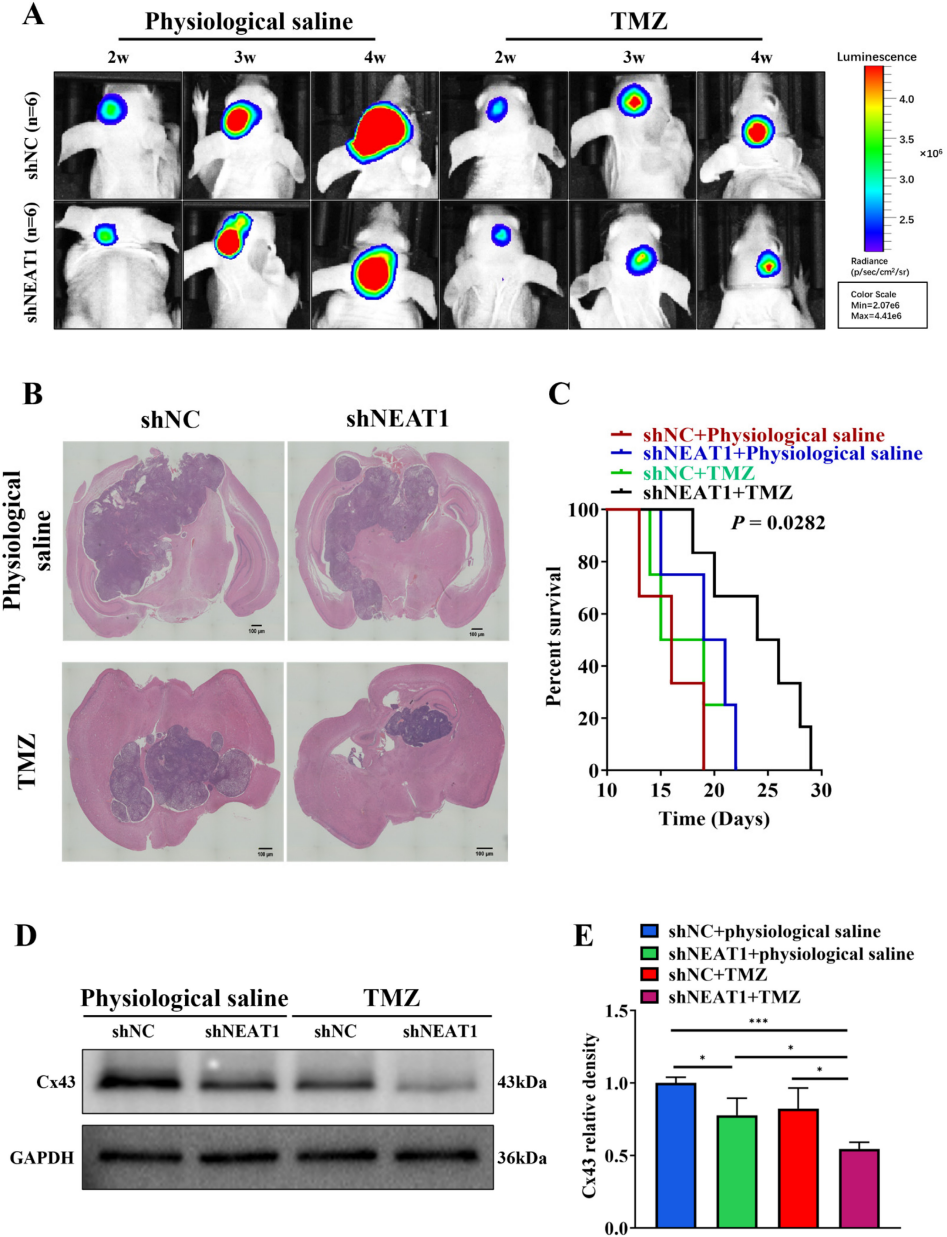
NEAT1 depletion enhances TMZ sensitivity in vivo. (A) Representative bioluminescence images of intracranial xenografts bearing U87MG‐Luc/shNC or U87‐Luc/shNEAT1 with TMZ (20 mg/kg) or same volume of physiological saline at indicated time points, *n* = 6. (B) Intracranial tumors in brain were shown by H&E staining. Scale bar = 100 μm. (C) Kaplan–Meier survival curve of nude mice is shown. (D, E) The total proteins were extracted from xenografts and subjected to Western blot analysis for Cx43 expression. GAPDH was the control. *p < 0.05; ***p < 0.001.

An experiment was conducted utilizing shNEAT1 in a tumor formation model to analyze the effect of NEAT1 on the regulation of TMZ chemosensitivity in a tumor formation animal model (*n* = 6). The shNEAT1 group exhibited a substantial enhancement in TMZ sensitivity in vivo, as evidenced by the data presented in Figure 5A,B. Furthermore, mice with decreased NEAT1 expression demonstrated prolonged survival compared to shNC mice when subjected to TMZ treatment, as illustrated in Figure 5C. Additionally, Figure 5D showed Cx43 protein expression levels were considerably reduced in the shNEAT1 group with TMZ treatment. These findings indicated that knockdown of NEAT1 contributes to increased sensitivity to TMZ in brain tumor.

## 4 Discussion

The purpose of this research was to examine how the lncRNA NEAT1 impacts chemo‐sensitivity in GBM. An increase in NEAT1 expression was observed in GBM samples, especially in recurrence samples, and these results were confirmed by an analysis of the CGGA database. Although NEAT1 has been shown to be upregulated in GBMs, study on NEAT1's molecular mechanism of promoting chemotherapy sensitivity in GBMs is limited [34].

Study by Xing et al. [35] found that long non‐coding RNAs may be responsible for increasing or decreasing the sensitivity of cancer cells to chemotherapy. Recent evidence suggested that lncRNAs function through multiple mechanisms of action, interacting directly with signal receptors, regulating transcription, epigenetic modifications, protein/RNA stability, interacting with DNA, RNA and/or proteins, and post‐translational modifications [36]. Within the cytoplasm, lncRNAs have the ability to sequester miRNA and proteins, thereby modulating their activity and abundance [37]. Additionally, lncRNAs can impact protein posttranslational modifications and facilitate mRNA translation and stability [38, 39]. Although NEAT1 is mainly found in the nucleus, we also detected its presence in the cytoplasm. By using bioinformatics predictions, double luciferase reporter gene assays, and RIP assays, miR‐454‐3p was identified as a target of NEAT1. It was apparent from the negative correlation between NEAT1 and miR‐454‐3p expression levels in clinical samples that they likely function as ceRNA. Overexpressed or silencing miR‐454‐3p resulted in decreased or elevated NEAT1 levels, suggesting that NEAT1 functions as a decoy for miR‐454‐3p at molecular level.

Using bioinformatics analysis, we discovered a possible connection between miR‐454‐3p and GJA1, a gene implicated in resistance to GBM. Using dual luciferase reporter gene assays and western blot analysis with miR‐454‐3p overexpressed and interfered with in order to confirm that GJA1 was the target of miR‐454‐3p. Our analysis of GBM tissues revealed a reverse relationship between GJA1 expression and miR‐454‐3p. Gap junctions (GJs) are necessary for cells, including cancer cells, to exchange ions, metabolites, and second messengers, to maintain cell survival [40, 41, 42]. Gap junctions created by Connexin 43 are frequently observed at the junctions between astrocytes and cells from different individuals. Blocking GJs prevents the transfer of essential compounds between cells and leads to apoptosis [43]. The deficiency of Cx43 causes gap junction intercellular communication (GJIC) dysfunction [44]. In our study, we found that silencing NEAT1 blocked GJIC, while Cx43 overexpression restored GJIC. Our results also showed that Cx43 overexpression reversed the NEAT1‐silencing‐regulated apoptosis‐related proteins BAX and BCL2. To sum up, silencing NEAT1 might block GJIC through regulating Cx43, thereby promoting cell sensitivity to TMZ.

In summary, our research findings suggest that NEAT1 exhibit elevated expression levels in gliomas, especially in cases that recurred, and TMZ effectively enhances the response of GBM cells to NEAT1 inhibition. Gap junctional intercellular communication (GJIC) is regulated by NEAT1 through its interaction with miR‐454‐3p, which targets Cx43. This ultimately impacts the response of GBM to TMZ chemotherapy. Consequently, we propose that the NEAT1/miR‐454‐3p axis significantly contributes to the emergence of chemotherapy resistance in GBM, and our study offers valuable insights into potential targets and developing therapeutic approaches for managing this aggressive brain tumor.

## Author Contributions

**Jinxing Liang:** validation, investigation, project administration, formal analysis, writing – original draft, data curation. **Jia‐xiu Xie:** investigation, validation, formal analysis. **Junhui He:** validation. **Yi Li:** validation. **Dongmei Wei:** project administration, formal analysis, validation. **Rongfei Zhou:** validation. **Guining Wei:** project administration. **Xuehua Liu:** resources. **Qiudan Chen:** formal analysis, funding acquisition, conceptualization, data curation. **Dongmei Li:** conceptualization, formal analysis, project administration, writing – original draft, writing – review and editing, funding acquisition, investigation.

## Ethics Statement

The research with human subjects underwent evaluation and received authorization from the medical ethics board at Sir Run Run Hospital of Nanjing Medical University. Through a written informed agreement, study participants consented to participate in this research. Ethics Committee on Laboratory Animal Management of Guangxi Institute of Chinese Medicine and Pharmaceutical Science approved the experiments (Approval Document No. 20211104).

## Conflicts of Interest

The authors declare no conflicts of interest.

## Supporting information


Data S1.

Figure S1.

## Data Availability

The data utilized and examined in the present study can be obtained upon request from the corresponding author.
