# Supplementary material for: Inhibiting lncRNA NEAT1 Increases Glioblastoma Response to TMZ by Reducing Connexin 43 Expression
Source: Cancer Rep (Hoboken). 2024 Oct 25;7(10):e70031. doi: 10.1002/cnr2.70031 (PMC11505515; doi:10.1002/cnr2.70031)
Supplement: Supplementary file 1 — Data S1. [file CNR2-7-e70031-s001.pptx]

## Slide 1
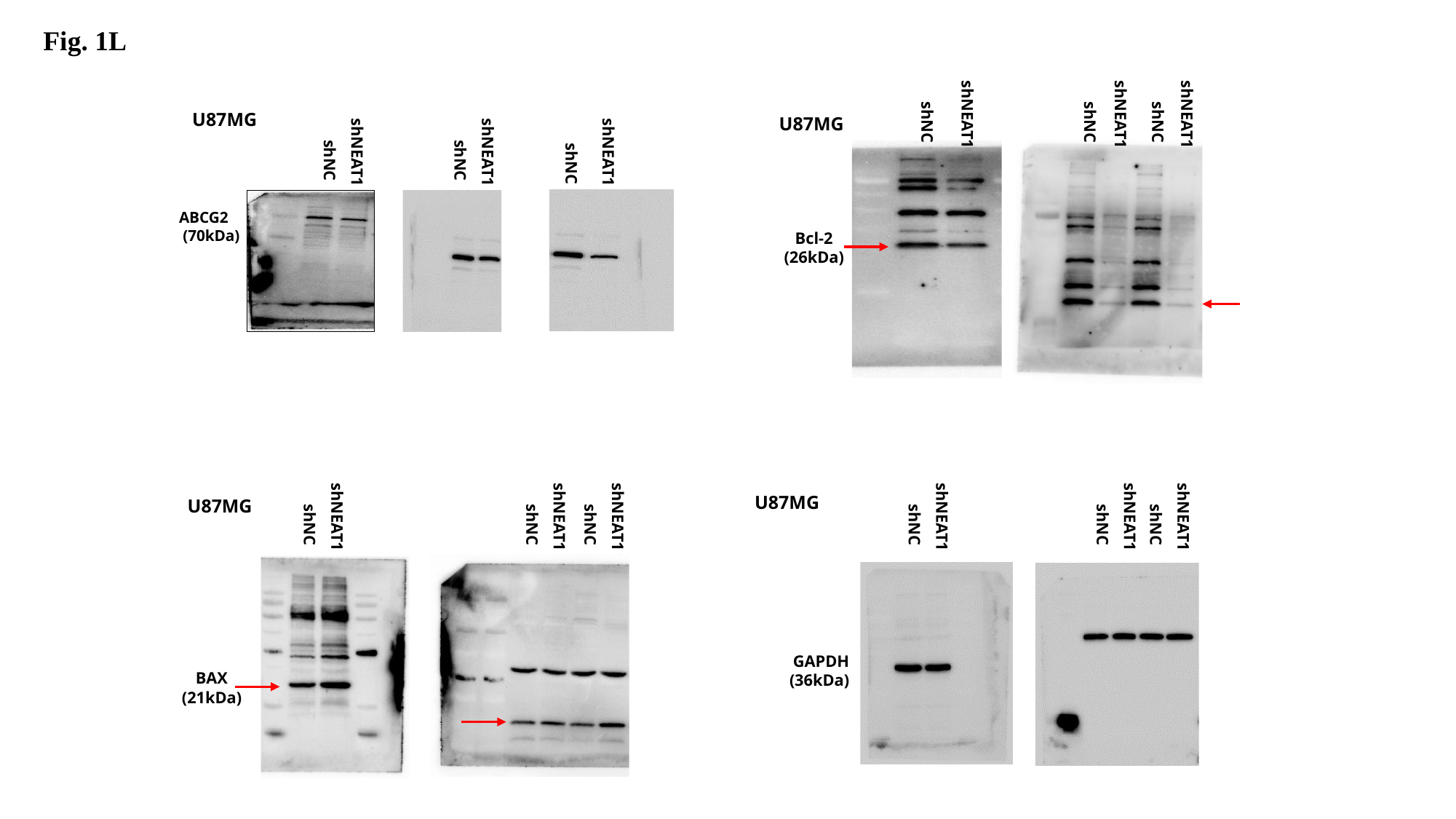

Fig. 1L
shNEAT1
shNC
shNEAT1
shNC
shNEAT1
shNC
U87MG
U87MG
shNEAT1
shNC
shNEAT1
shNC
shNEAT1
shNC
ABCG2
 (70kDa)
Bcl-2
(26kDa)
shNEAT1
shNC
shNEAT1
shNC
shNEAT1
shNC
shNEAT1
shNC
shNEAT1
shNC
shNEAT1
shNC
U87MG
U87MG
GAPDH
(36kDa)
BAX
(21kDa)

## Slide 2
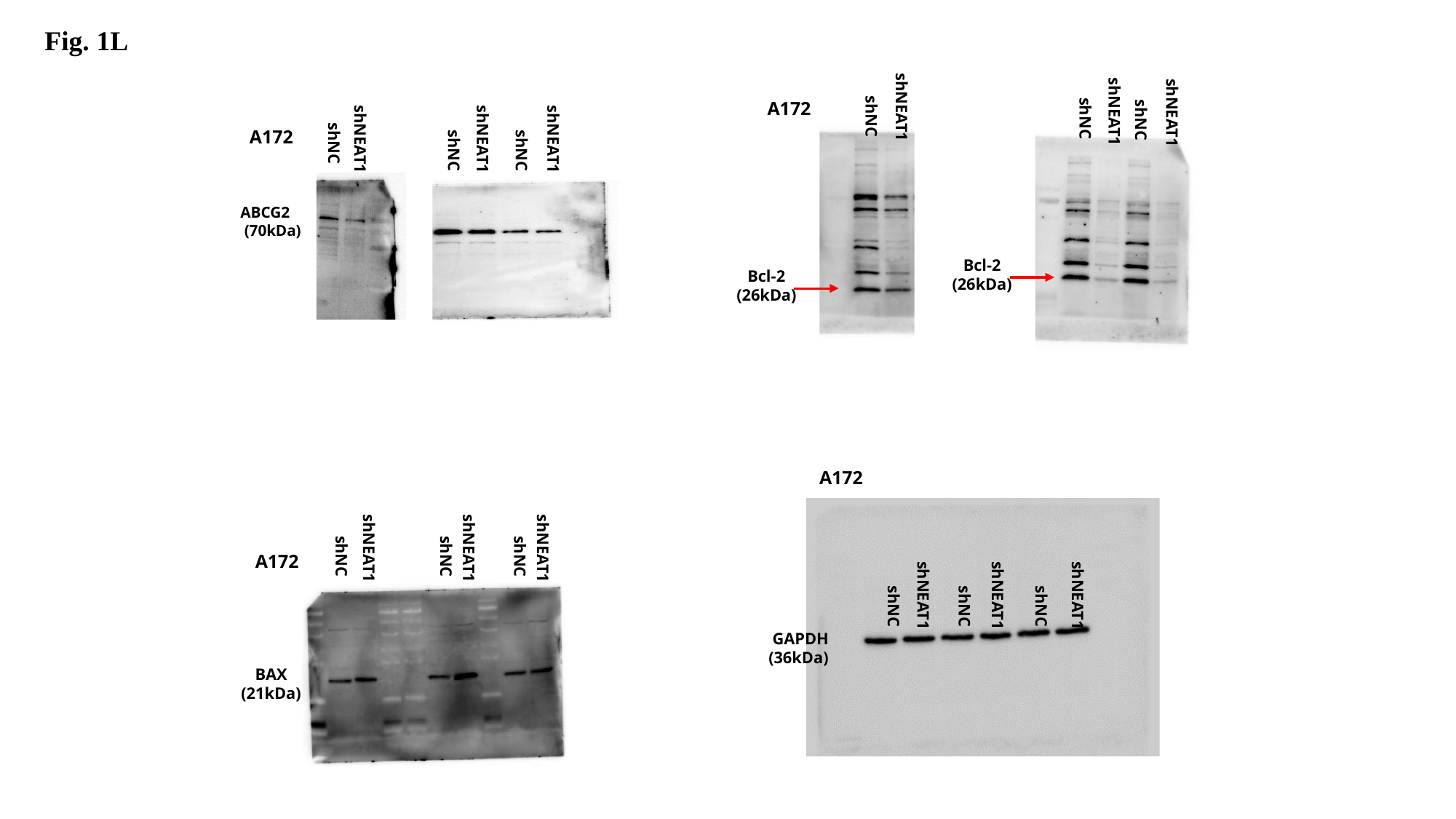

Fig. 1L
shNEAT1
shNEAT1
shNEAT1
shNC
shNC
shNC
A172
Bcl-2
(26kDa)
Bcl-2
(26kDa)
shNEAT1
shNC
shNEAT1
shNEAT1
shNC
shNC
A172
ABCG2
 (70kDa)
A172
shNEAT1
shNEAT1
shNEAT1
shNC
shNC
shNC
GAPDH
(36kDa)
shNEAT1
shNC
shNEAT1
shNC
shNEAT1
shNC
A172
BAX
(21kDa)

## Slide 3
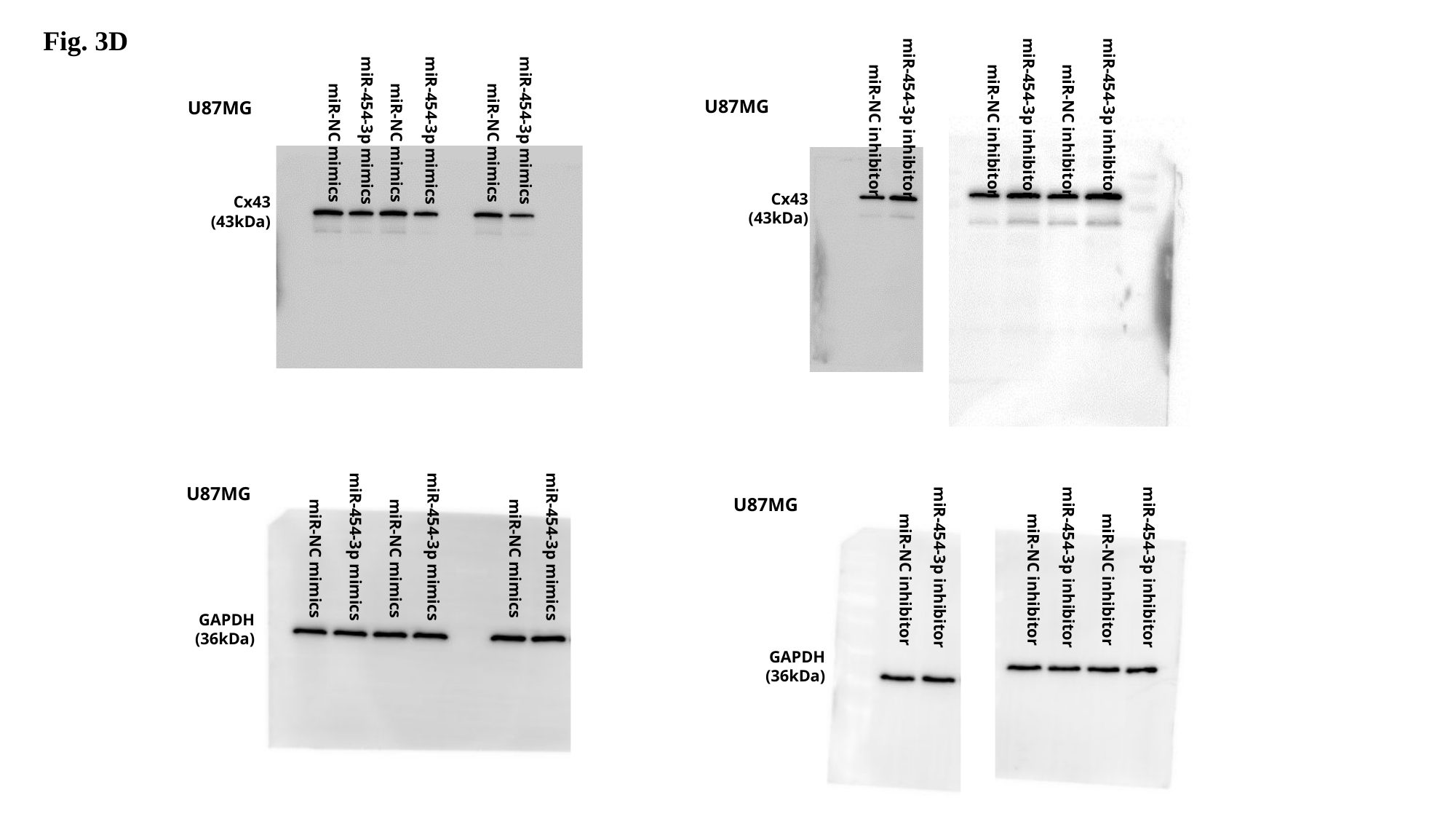

Fig. 3D
miR-454-3p inhibitor
miR-454-3p inhibitor
miR-454-3p inhibitor
miR-454-3p mimics
miR-454-3p mimics
miR-454-3p mimics
miR-NC inhibitor
miR-NC inhibitor
miR-NC inhibitor
miR-NC mimics
miR-NC mimics
miR-NC mimics
U87MG
U87MG
Cx43
(43kDa)
Cx43
(43kDa)
miR-454-3p mimics
miR-454-3p mimics
miR-454-3p mimics
miR-454-3p inhibitor
miR-454-3p inhibitor
miR-454-3p inhibitor
U87MG
miR-NC mimics
miR-NC mimics
miR-NC mimics
U87MG
miR-NC inhibitor
miR-NC inhibitor
miR-NC inhibitor
GAPDH
(36kDa)
GAPDH
(36kDa)

## Slide 4
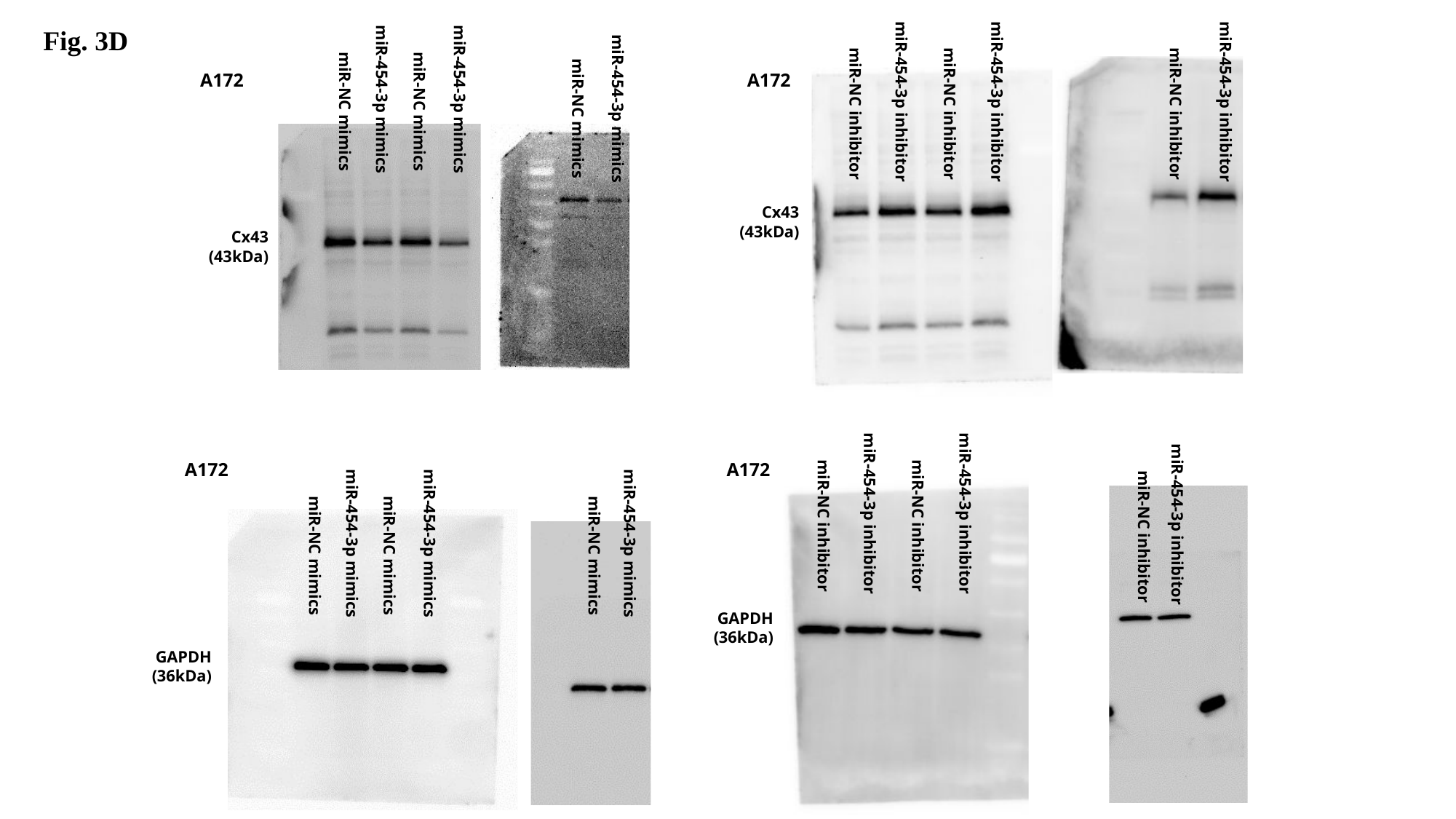

miR-454-3p inhibitor
miR-454-3p inhibitor
miR-454-3p inhibitor
miR-454-3p mimics
miR-454-3p mimics
miR-454-3p mimics
miR-NC mimics
miR-NC mimics
miR-NC mimics
A172
Cx43
(43kDa)
Fig. 3D
miR-NC inhibitor
miR-NC inhibitor
miR-NC inhibitor
A172
Cx43
(43kDa)
miR-454-3p inhibitor
miR-454-3p inhibitor
miR-454-3p inhibitor
miR-NC inhibitor
miR-NC inhibitor
A172
A172
miR-454-3p mimics
miR-454-3p mimics
miR-454-3p mimics
miR-NC inhibitor
miR-NC mimics
miR-NC mimics
miR-NC mimics
GAPDH
(36kDa)
GAPDH
(36kDa)

## Slide 5
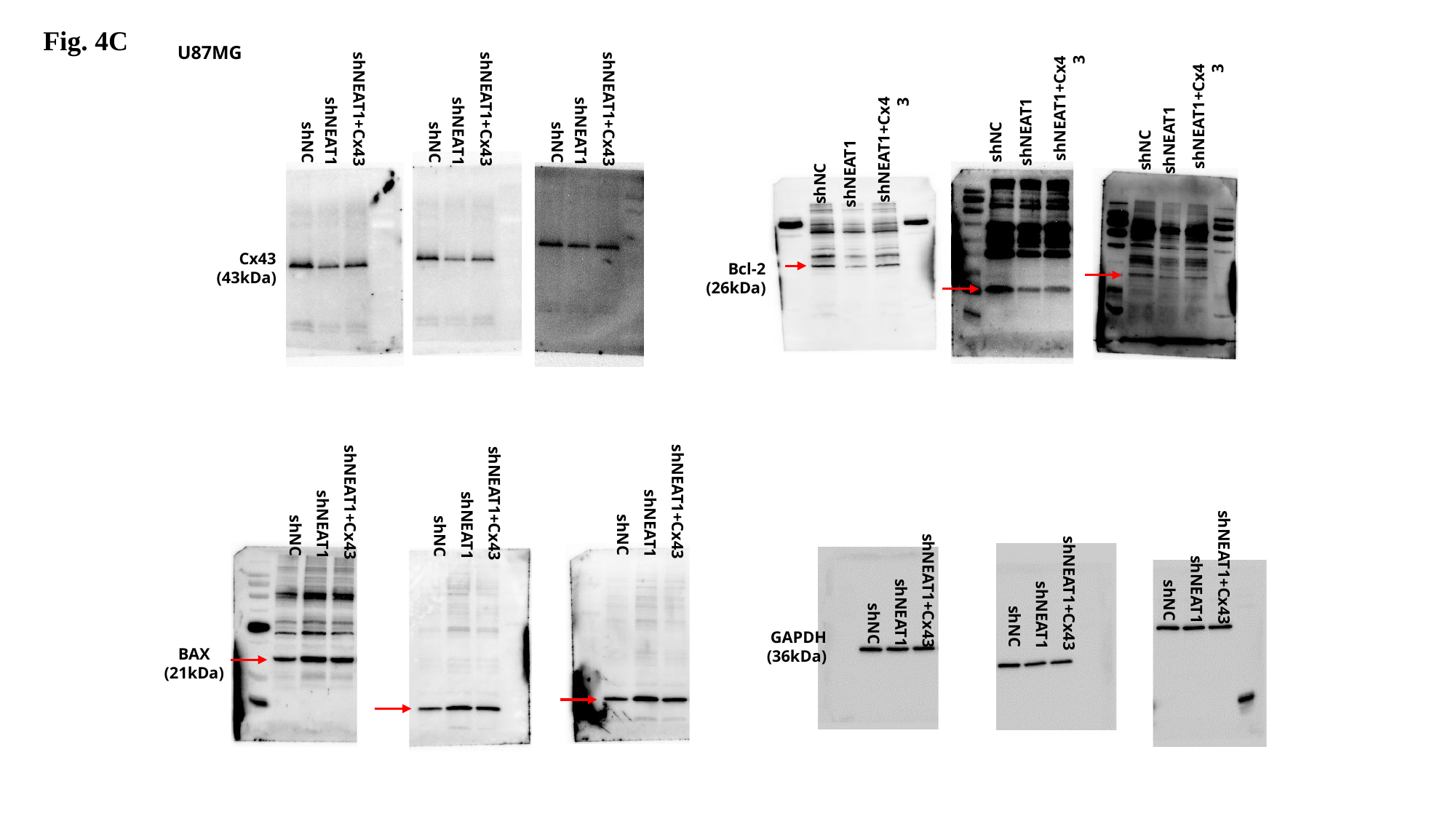

Fig. 4C
U87MG
shNEAT1+Cx43
shNEAT1
shNC
shNEAT1+Cx43
shNEAT1
shNC
shNEAT1+Cx43
shNEAT1
shNC
Cx43
(43kDa)
shNEAT1+Cx43
shNEAT1
shNC
shNEAT1+Cx43
shNEAT1
shNC
shNEAT1+Cx43
shNEAT1
shNC
Bcl-2
(26kDa)
shNEAT1+Cx43
shNEAT1
shNC
shNEAT1+Cx43
shNEAT1
shNC
shNEAT1+Cx43
shNEAT1
shNC
BAX
(21kDa)
shNEAT1+Cx43
shNEAT1
shNC
shNEAT1+Cx43
shNEAT1
shNC
shNEAT1+Cx43
shNEAT1
shNC
GAPDH
(36kDa)

## Slide 6
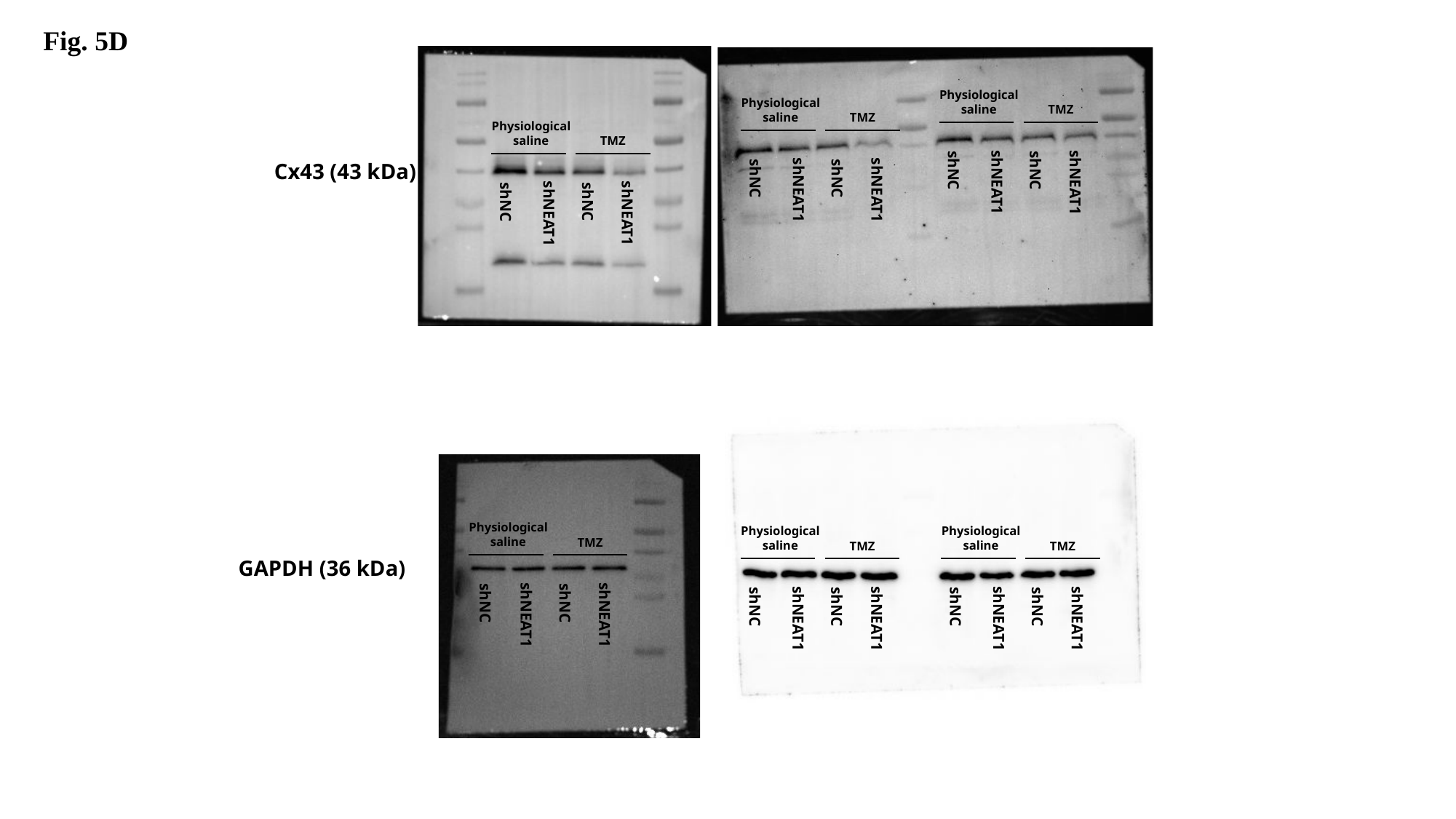

Fig. 5D
Physiological saline
TMZ
shNC
shNC
shNEAT1
shNEAT1
Physiological saline
TMZ
shNC
shNC
shNEAT1
shNEAT1
Physiological saline
TMZ
shNC
shNC
shNEAT1
shNEAT1
Cx43 (43 kDa)
Physiological saline
TMZ
shNC
shNC
shNEAT1
shNEAT1
Physiological saline
TMZ
shNC
shNC
shNEAT1
shNEAT1
Physiological saline
TMZ
shNC
shNC
shNEAT1
shNEAT1
GAPDH (36 kDa)
