# Supplementary material for: Inhibiting lncRNA NEAT1 Increases Glioblastoma Response to TMZ by Reducing Connexin 43 Expression
Source: Cancer Rep (Hoboken). 2024 Oct 25;7(10):e70031. doi: 10.1002/cnr2.70031 (PMC11505515; doi:10.1002/cnr2.70031)
Supplement: Supplementary file 2 — Figure S1. [file CNR2-7-e70031-s002.docx]

**Supplementary**


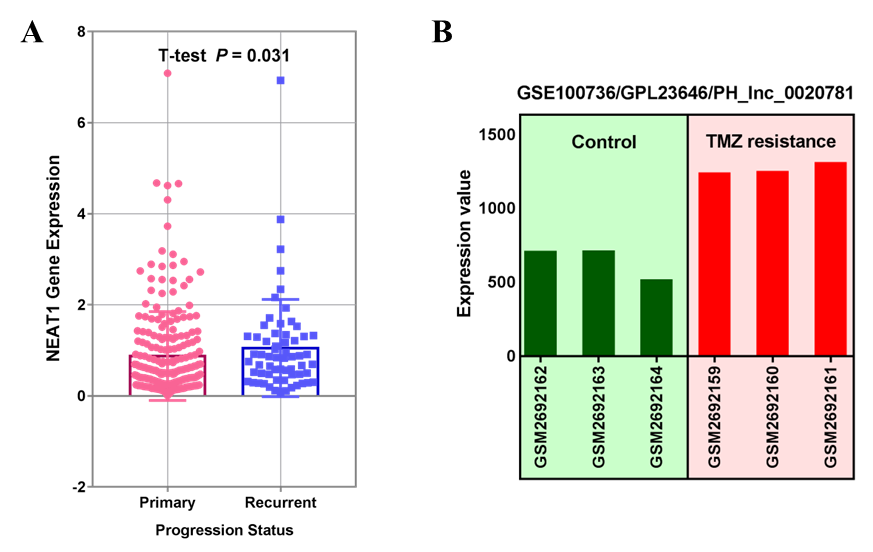


**Figure S1** (A)The expression of NEAT1 in primary and recurrent glioma patient samples in the CGGA. (B) The expression of NEAT1 in GSE100736 database.
